# Poly(Cyclohexene Phthalate) Nanoparticles for Controlled Dasatinib Delivery in Breast Cancer Therapy

**DOI:** 10.3390/nano9091208

**Published:** 2019-08-27

**Authors:** Enrique Niza, Cristina Nieto-Jiménez, María del Mar Noblejas-López, Iván Bravo, José Antonio Castro-Osma, Felipe de la Cruz-Martínez, Marc Martínez de Sarasa Buchaca, Inmaculada Posadas, Jesús Canales-Vázquez, Agustín Lara-Sanchez, Daniel Hermida-Merino, Eduardo Solano, Alberto Ocaña, Carlos Alonso-Moreno

**Affiliations:** 1 Dpto. Inorgánica, Orgánica y Bioquímica. School of Pharmacy, University of Castilla-La Mancha, 02071 Albacete, Spain; 2 Oncología traslacional, Centro Regional de Investigaciones Biomédicas, University of Castilla-La Mancha, 02071 Albacete, Spain; 3 Dpto. Química Física. School of Pharmacy, University of Castilla-La Mancha, 02071 Albacete, Spain; 4 Dpto. Inorgánica, Orgánica y Bioquímica. Facultad de Ciencias y Tecnologías Químicas, University of Castilla-La Mancha, 13075 Ciudad Real, Spain; 5 Unidad Asociada Neurodeath CSIC-UCLM, Dpto. de Ciencias Médicas, School of Pharmacy, University of Castilla-La Mancha, 02071 Albacete, Spain; 6 Instituto de Energías Renovables, University of Castilla-La Mancha, 02071 Albacete, Spain; 7 Netherlands Organisation for Scientific Research (NWO), DUBBLE@ESRF, 38000 Grenoble, France; 8 NCD beamline, ALBA Synchrotron Light Source, Carrer de la Llum 2-26, 08290 Cerdanyola del Vallès, Spain; 9 Hospital Clínico San Carlos, 28036 Madrid, Spain

**Keywords:** dasatinib, breast cancer, poly(cyclohexene phthalate), polymeric nanoparticles

## Abstract

The effect on the activity in breast cancer models of the small tyrosine kinase inhibitor dasatinib (DAS), either alone or in combination with other antitumoral agents, has been recently explored. However, DAS is characterized by its low and highly pH-dependent solubility, which could lead to poor uptake of the drug limiting its tumoral efficacy. Thus far, the development of safe and efficient delivery vehicles of DAS to improve the therapeutic efficacy minimizing the toxicity profile is still required. In this work, a biodegradable and biocompatible polyester is assessed, for the first time, as raw material for the generation of polymeric nanoparticles (NPs). NPs of 100 nm with a narrow polydispersity were formulated for the encapsulation of DAS. The enzymatic and cellular degradation of the new drug delivery system has been studied, and the toxicity and blood compatibility evaluated for its potential clinical use. The new material used for the generation of nanoparticles led to encapsulate DAS in an efficient manner with quicker release DAS profile when compared with the FDA-approved biopolymer Polylactide. The new DAS-loaded polymeric nanocarrier gave a superior efficacy when compared to free DAS with no difference in the mechanism of action. The new NPs shown to be a promising DAS delivery system to be further evaluated for breast cancer treatment.

## 1. Introduction

In addition to the importance of identifying new therapeutic targets, the development of novel Drug Delivery Systems (DDS) is a therapeutic strategy to pursuit to increase efficacy and reduce toxicity of well characterized molecules [1,2]. Several macromolecular structures are being explored as DDS [3,4]. Among them, polymeric nanoparticles (NPs) are considered one of the most promising nanocarriers for use in oncology [5,6,7,8,9,10]. Their building blocks are biodegradable and biocompatible, and their release profiles can be modulated by the polymeric structure itself. Polyesters derived from glycolide, lactone and lactide and their copolymers show biodegradability and biocompatibility properties and are commonly used as raw materials for DDS preparation [11,12]. In fact, there are commercially available DDS based on polyesters [13].

Common ways to obtain biodegradable and biocompatible polyesters via polycondensation processes or physical aggregation have important limitations, such as the control of the molecular weight, the increase on polydispersity values or the non-existent control over the stereoregularity of the polymer. The physicochemical properties of polymeric NPs as DDS, such as size, molecular weight, molecular weight distributions, crystallinity and polarity will determine their efficacy and viability. Organometallic catalysis allows the preparation of polymers and copolymers with a precise control in their structure. Currently, aliphatic polyesters such as polycaprolactone and polylactide can be catalytically produced via ring-opening polymerization [12]. Nevertheless, the available number of polyesters obtained by this strategy is seriously restricted by the limited number of commercially available cyclic esters. As an alternative, new promising strategies to get polyesters based on catalysis have been explored, such as ring-opening copolymerization (ROCOP) of epoxides with anhydrides [14,15]. 

Dasatinib (DAS) is a tyrosine kinase inhibitor able to target various kinases such as Src, c-Kit and others [16,17,18]. It is an FDA-approved multi-target compound for the treatment of chronic myeloid leukemia. DAS has become a potential candidate for the treatment of other cancers, such as lung, prostate and ovarian cancer [19,20,21]. In fact, the relevant role of DAS in HER2 positive breast cancers has been recently demonstrated in breast cancer therapy [22,23,24,25]. However, the solubility of DAS is extremely low and highly pH-dependent according to Biopharmaceutics Classification System (BCS). Its clinical use is also hampered by adverse side effects. The development of safe and efficient delivery vehicles of DAS could potentially improve the therapeutic efficacy minimizing the systemic toxicities. 

To date, there are not many examples reported for the encapsulation of DAS. Among them, polymeric micelles have been the most studied DAS carriers leading to a significantly inhibition of the tumor cell proliferation when compared to free DAS [26,27,28]. Albumin nanoparticles were also assessed as carriers to reduce DAS-induced endothelial hyperpermeability improving the anti-leukemia efficacy [29]. Layered polymer-coated carbon nanotubes were evaluated for the controlled delivery of DAS to enhance the in vitro efficacy against U-87 glioblastoma cells [30]. Very recently, magnetic protein micelles were reported to enhance delivery of DAS to human triple-Negative Breast cancer cells [31].

Herein, we present the formulation, characterization, citoxicity, biocompatibility and enzymatic degradation of polymeric nanoparticles made from poly(cyclohexene phthalate). The assessment of DAS-polymeric nanoparticles to improve breast cancer therapy is also discussed throughout the manuscript.

## 2. Materials and Methods

### 2.1. Preparation of CHO/PA NPs

#### 2.1.1. Materials

Tetrahydrofuran (THF for HPLC > 99%), Dimethyl sulfoxide (DMSO, 99.9%), Phosphate buffered saline (PBS, pH 7.4) and Poly(vinyl) alcohol (PVA, 31,000–50,000, 98–99% hydrolyzed) were purchased from Sigma-Aldrich Spain. Dasatinib Monohydrate (DAS) was purchased from Selleckchem (Madrid, Spain).

#### 2.1.2. Formulation

Poly(cyclohexene phthalate) (CHO/PA) NPs were prepared by nanoprecipitation. Briefly, 20 mg of CHO/PA was dissolved in 3 mL of THF and added dropwise (2 mL⋅min^−1^) to the aqueous solution (PVA 0.2%). The single emulsion was stirred for 5 min. The organic solvent from the resulting emulsion (O/W) was removed under reduced pressure. NPs were collected by centrifugation at 12,000× *g*, washed with deionized water (DI) and freeze-dried.

### 2.2. Preparation of DAS-Loaded CHO/PA NPs

#### 2.2.1. Formulation

DAS-loaded CHO/PA NPs were prepared by nanoprecipitation. Briefly, 3 mg of DAS were dissolved in 50 µl DMSO. The DAS solution was mixed with 20 mg of CHO/PA in 3 mL of THF and added dropwise (2 mL⋅min^−1^) to the aqueous solution (PVA 0.2%). The single emulsion was stirred for 5 min. The organic solvent from the resulting emulsion (O/W) was removed under reduced pressure. DAS-loaded NPs were collected by centrifugation at 12,000× *g*, washed with DI and freeze-dried. To calculate the loading efficiency, 1 mL of DMSO was added to NPs to dissolve both polymer and drug and the mixture was subjected to bath sonication for 30 min. DAS loading was measured in a spectrophotometer at 324 nm. Loading efficiency (LE) and encapsulation efficiency (EE) of DAS were calculated as follows:

LE = (weight of encapsulated DAS (mg))/(weight of total (DAS encapsulated + scaffold weight)(mg)) × 100%

EE = (weight of encapsulated DAS (mg))/(weight of DAS feeding (mg)) × 100%

#### 2.2.2. Stability of the CHO/PA NPs in Simulated Physiological Media

The stability of the DAS-loaded CHO/PA NPs was performed in 10% human blood plasma. Briefly, the DAS-loaded CHO/PA NPs were incubated at 37 °C, at a concentration equal to 1 mg·mL^−1^. The hydrodynamic radius (R_H_) and polydispersity index (PdI) of the formulations were calculated at predetermined intervals of time by Dynamic Light Scattering (DLS) measurements (University of Castilla-La Mancha, Albacete, Spain). 

### 2.3. Biological Studies

#### 2.3.1. Enzymatic Degradation Studies

The degradation of the CHO/PA NPs was studied in enzymatic and non-enzymatic medium [32,33]. Briefly, 1 mL of NPs suspension (10 mg⋅mL^−1^) was loaded into 5 mL tube and the volume adjusted to 5 mL with Phosphate Buffered Saline (PBS). 1.1 µg·mL^−1^ of lipase were added and incubated at 37 °C in the dark. DLS analysis was performed at predetermined intervals of time.

#### 2.3.2. In vitro Drug Release

1 mg of DAS devices (DAS-loaded CHO/PA NPs or DAS-loaded Polylactide NPs) was suspended in 25 mL of PBS buffer solution (pH 7.4). After incubation the samples at 37 °C, 3 mL of release medium was taken out and centrifuged at certain intervals. DAS-release concentration was measured in a spectrophotometer at 324 nm. Fresh release medium was added to the original release medium to maintain sink conditions. The drug releases were tested in three replicates.

#### 2.3.3. Blood Compatibility

Heparinized human blood was obtained from the Blood Donor Center of the University Hospital of Albacete (CHUA). Red blood cells (RBCs) were isolated by centrifugation at 350× *g* for 10 min at 4 °C. The pellet was rinsed three times with PBS (pH 7.4) and suspended in saline (0.9% sodium chloride). NPs, DAS-loaded NPs and DAS at different concentrations (200–800 nM of DAS and DAS-loaded NPs, and 20–80 µg·mL^−1^ of empty NPs) were added to 500 μL of erythrocyte suspension and incubated for three hours at 37 °C under constant shaking. PBS and 1% Triton w/v were added to erythrocyte suspension individually as negative and positive control, respectively. After three hours, the mixture was centrifuged, and the supernatant was analyzed for percent hemolysis by UV–visible photometry at 540 nm. Hemolysis rate was calculated as follows:

Hemolysis rate (%) = [(ODt-ODnc)/(ODpc-ODnc)] × 100

where ODt, ODnc and ODpc are the optical density (OD) values of the test samples, negative control group and positive control group, respectively.

#### 2.3.4. Cell Culture

Human embryonic kidney 293 cells (HEK-293) were grown in Roswell Park Memorial Institute medium supplemented with 10% heat-inactivated fetal calf serum. Cells were maintained at 37 °C in a saturated humidity atmosphere (95% air and 5% CO_2_). MDA-MB-231, HS578T and BT549 were grown in Dulbecco’s Modified Eagle medium (DMEM) supplemented with 10% heat-inactivated fetal bovine serum. MCF10A were cultured with DMEM F12 medium supplemented with EGF (20 ng mL^−1^), hydrocortisone (0.5 mg·mL^−1^), insulin (10 μg·mL^−1^) and 5% heat-inactivated horse serum. All mediums were supplemented with 2 mM L-glutamine, penicillin (20 units·mL^−1^) and streptomycin (5 μg·mL^−1^). Cells were maintained at 37 °C in a saturated humidity atmosphere containing 95% air and 5% CO_2_. 

#### 2.3.5. Toxicity

MTT assays were performed as described previously [34]. Non-tumoral cells (HEK-293, MDA-MB-231, HS578T, BT549 and MCF10A) were seeded onto 24-well plates and maintained at 37 °C in a saturated humidity atmosphere containing 95% air and 5% CO_2_ until 80% of confluence was reached. Afterwards, non-tumoral cells (HEK-293 and MCF10A) were treated with vehicle (PBS) or 0.1 mg·mL^−1^ CHO/PA for 24, 48 and 72 h, whereas tumoral cells (MDA-MB-231, HS578T and BT549) were treated with vehicle, DAS or DAS-loaded CHO/PA NP for 24, 48 and 72 h. MTT (5 mg·mL^−1^) was subsequently added to each well and the cells were incubated at 37 °C for 1 h. The culture medium was removed, and the insoluble formazan crystals were dissolved in 300 μL DMSO and aliquots of 200 μL were transferred to a 96-well microplate and measured spectrophotometrically in an ELISA reader at 590 nm.

#### 2.3.6. Cell Cycle Studies

MDA-MB-231, HS578T and BT549 were seeded onto 24-well plates and maintained at 37 °C in a saturated humidity atmosphere containing 95% air and 5% CO_2_ until 80% of confluence was reached. Afterwards, cells were treated with vehicle (PBS), DAS or DAS-loaded CHO/PA NPs for 24 h. Afterwards, cells were scrapped, centrifuged at 500× *g* for 10 min to remove medium and fixed with 70% cold ethanol for 30 min. Then, cells were washed twice and stained with propidium iodide/RNAse staining solution (Immunostep S.L.). Vehicle-treated cells were used as control. Results were analyzed on FACSCanto II flow cytometer (BD Biosciences). The percentage of cells in each cell-cycle phase was determined by plotting DNA content against cell number using the FACS Diva software (BD Biosciences, CA, US).

#### 2.3.7. Apoptosis

MDA-MB-231, HS578T and BT549 were seeded onto 24-well plates and maintained at 37 °C in a saturated humidity atmosphere containing 95% air and 5% CO_2_ until 80% of confluence was reached. Afterwards, cells were treated with vehicle (PBS), DAS or DAS-loaded CHO/PA NPs for 72 h. Then, they were collected and stained in the dark with Annexin V-DT-634 (Immunostep S.L.), and propidium iodide at room temperature for 1 h. Apoptotic cells were determined using a FACSCanto II flow cytometer (BD Biosciences). 

## 3. Results and Discussion

### 3.1. Synthesis and Characterization of Raw Material

**Scheme 1:**
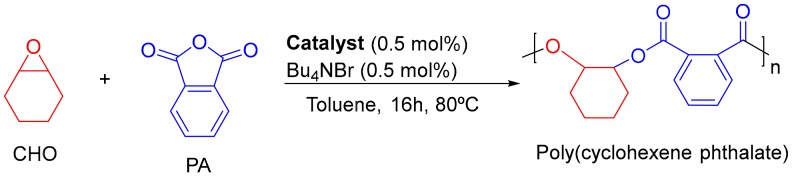
ROCOP of cyclohexene oxide (CHO) and phthalic anhydride (PA) catalyzed by aluminium compounds.

Poly(cyclohexene phthalate) (CHO/PA) was obtained by Ring-Opening Copolymerization (ROCOP) of epoxides and cyclic anhydrides as reported previously (Scheme 1) [35]. Table 1 shows 100% of conversion towards polyesters. Copolymers were carefully washed until SEM-EDX analysis showed no residues of Al catalyst [36]. Microstructural analysis of the copolymer obtained was carried out by nuclear magnetic resonance spectroscopy (see Figure S1 in Supplementary Materials) and size exclusion chromatography [35].

### 3.2. Preparation and Characterization of CHO/PA NPs

**Figure 1:**
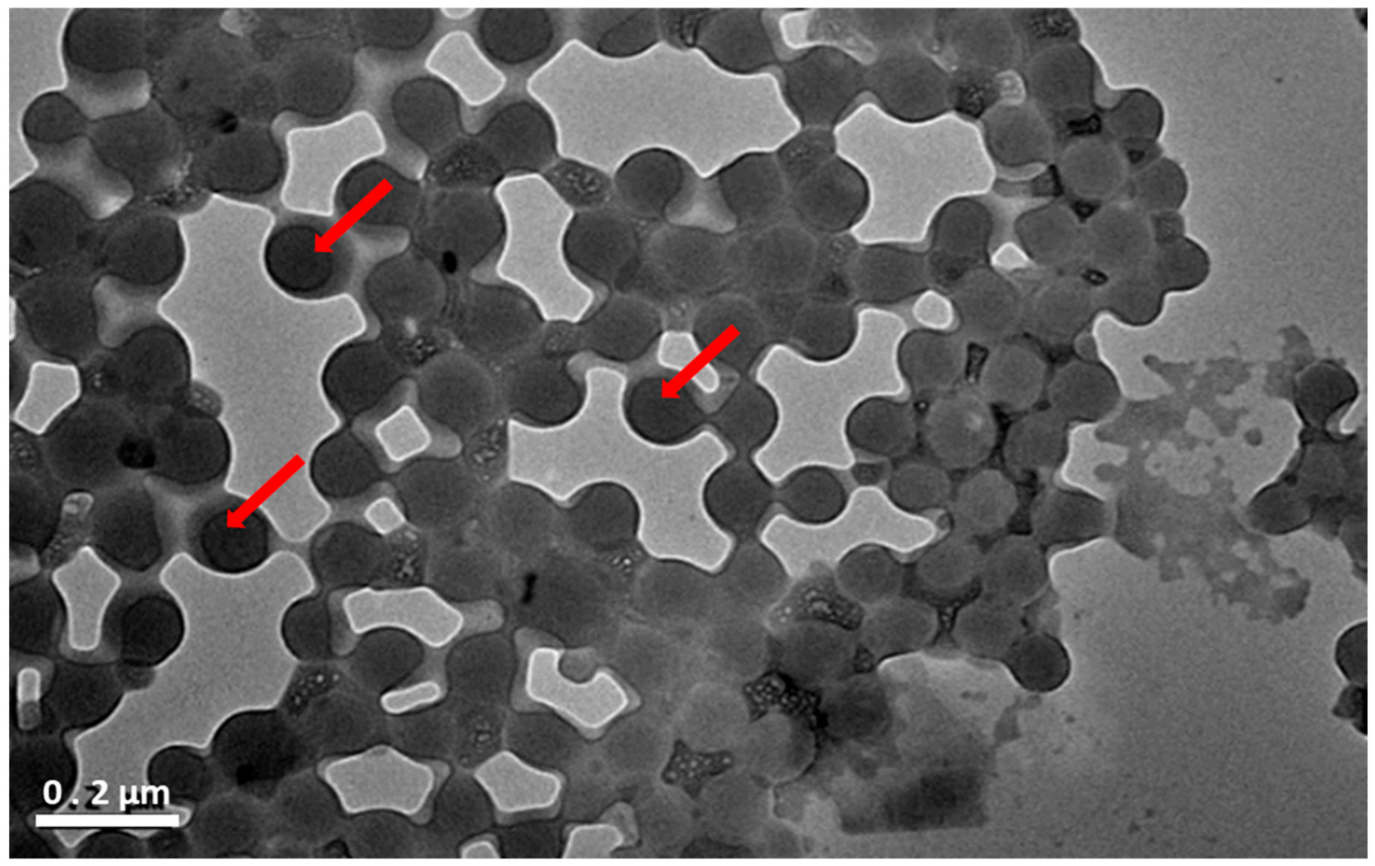
Transmission electron microscopy (TEM) images of CHO/PA NPs made from CHO/PA copolymer (Entry 3, Table 2). The TEM micrograph reveals that the nanoparticles surface is not perfectly flat as suggested by the combination of bright and dark (marked with arrows) contrast areas.

Nanoprecipitation were selected for CHO/PA formulation because it is one of the most effective methodology for the encapsulation of hydrophobic drugs [37]. Several variables were explored for optimizing formulation according to standardized procedures, such as surfactants (Polaxamer and PVA), surfactant concentration (0.2–1%) and the use of different organic solvents (THF and acetone). PVA (0.2%) and THF gave rise the best formulation parameters. Once optimized the NPs formulation, CHO/PA of different molecular weight (Table 1) were employed as raw materials to produce NPs with a monodisperse size distribution using nanoprecipitation methodology (see procedure in Materials and Methods). Thermal analysis, electron microscopy, SAXS/WAXS experiments, and hydrodynamic diameter and Z-potential measurements were carried out for characterization of the NPs. The size distribution, average size and *Z*-potential of nanocarriers are listed in Table 2. DLS showed unimodal size distributions with small PdI and negative *Z*-potential values. The particle size as well as morphological study of the CHO/PA NPs was observed by transmission electron microscopy (TEM) (Figure 1). NPs showed homogeneous spherical shapes (100–150 nm) with a rough surface as can be clearly appreciated by TEM (Figure 1). Overall, the results indicated that the molecular weight under study did not directly influence in the stability and size of the resulting nanoparticles.

The thermal stability levels of NPs were determined by thermogravimetric analysis (TGA). CHO/PA NPs were stable from 10 to 150 °C (see Figure S2 in Supplementary Materials). As expected, NPs showed similar thermal degradation patterns when compared to the raw material indicating no significant physicochemical changes in the polymer structure after formulation. The thermal properties of the NPs were investigated by differential scanning calorimetry (DSC). The second heating endotherms for the CHO/PA NPs in comparison with the raw materials are shown in Figure S3 in the Supplementary Materials. A single T_g_ has been observed at 108.9 °C and 121.9 °C for the raw material and the CHO/PA NP, respectively. The nanostructure of the NPs was further investigated by small and wide-angle scattering experiments (SAXS/WAXS). Comparison between CHO/PA NPs and raw materials are shown in Figure S4 in the Supplementary Materials. SAXS profiles indicated the lack of well-structured mesophase that would be expected as a result of the low molecular weight and the miscibility of the copolymer. Moreover, the WAXS patterns have shown a lack of crystallinity of the polymers in good agreement with DSC results. Both experiments showed that there was not any change into crystallinity of the copolymer after formulation.

### 3.3. Enzymatic Degradation of CHO/PA NPs

**Figure 2:**
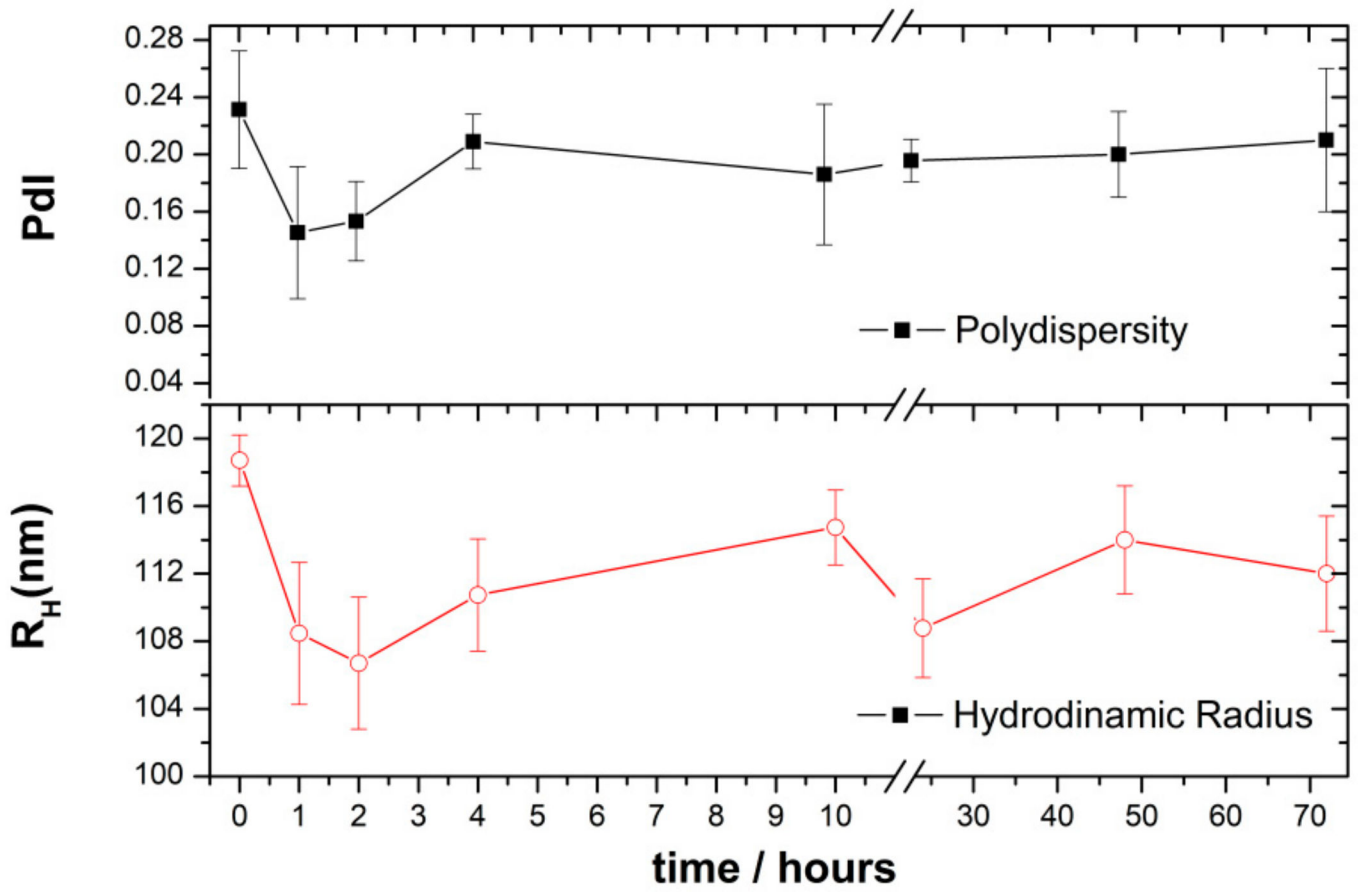
Enzymatic degradation of CHO/PA NPs in the presence of porcine lipase by DLS analysis. Data are expressed as mean ± s.e.m. from at least three independent experiments.

The enzymatic degradation of CHO/PA NPs in the presence of pancreatic lipase from porcine pancreas was evaluated by DLS analysis [32,33]. CHO/PA NPs were incubated with lipases along the time (see procedure in Materials and Methods). In the DLS experiments, PdI and hydrodynamic radius (R_H_) of NPs were measured with the incubation time in the presence of lipase (Figure 2). The negligible changes on the R_H_ and PdI of the NPs suggested that the degradation process is slow. The slight decrease of R_H_ and PdI values within the first 2 h may indicate a quick action of the lipases. However, the size increases from this point up to 4 h and remains almost stable up to 24 h. This suggests the formation of a protein layer (~8 nm) onto the NP surface after 2 h that presumably self-protect from the degradation. No significant changes were observed in R_H_ and PdI after 72 h without the presence of the enzyme.

### 3.4. CHO/PA NPs Toxicity in Non-Tumoral Cells

**Figure 3:**
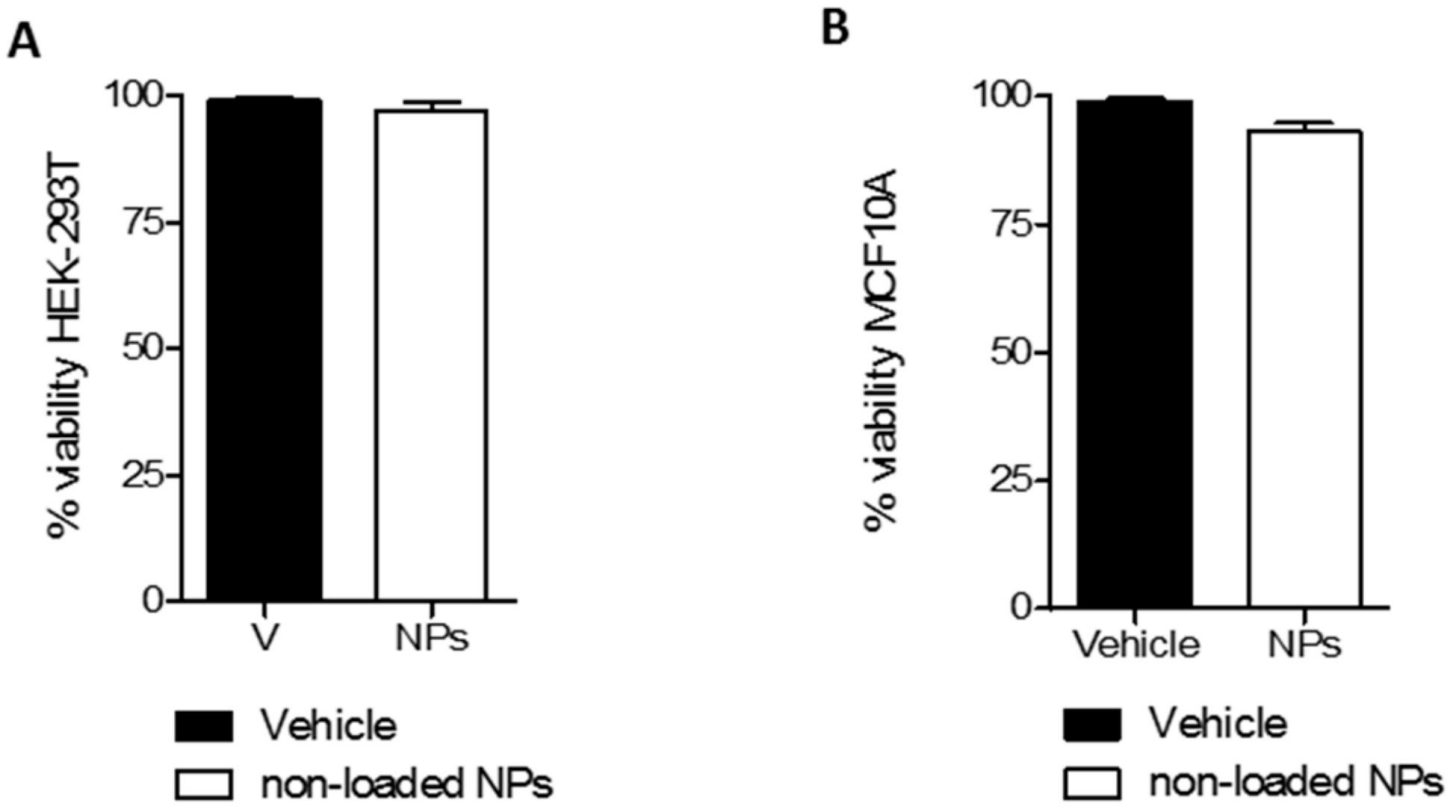
Cytotoxic effect of non-loaded CHO/PA NPs (0.1 mg·mL^−1^). (**A**). In human embryonic kidney 293 (HEK-293) cells. (**B**). In MCF10A cells. Data are expressed as mean ± s.e.m. from at least three independent experiments.

The cytotoxic effect on non-tumoral cells is a key factor that determines the safety and applicability of new materials. The HEK-293 cell line and the MCF10A cell line were selected for in vitro cytotoxicity evaluation of CHO/PA NPs in non-tumoral human cells. The cytotoxicity was determined using the MTT assay (Figure 3). Remarkably, non-loaded CHO/PA NPs did not display toxicity in the non-tumoral cells used as models, indicating an appropriate biosecurity profile of the NPs in study. The concentration of CHO/PA NPs selected to perform this study (0.1 mg·mL^−1^) was slightly higher than the nanoparticles present in DAS-loaded CHO/PA NPs formulation. The absence of toxicity at this concentration suggests good biocompatibility of lower concentrations of CHO/PA NPs with non-tumoral cells.

### 3.5. DAS Loading and Release

The viability of CHO/PA NPs as carrier of DAS for cancer therapy was carried out. Stable DAS-loaded nanoemulsions were obtained by nanoprecipitation. The optimized formulation consisted in the dropwise addition of organic solution of CHO/PA and DAS to the aqueous solution containing PVA (0.2%). The single emulsion was stirred for 5 min, the organic solvent removed under reduced pressure and NPs collected by centrifugation at 12,000× *g*, washed with DI and freeze-dried. The encapsulation of DAS into CHO/PA NPs did not cause significant changes in their size (see Table 2 and Table 3). To discuss the viability of CHO/PA NPs as drug carrier, commercially available Poly(L-Lactide) (PLA) (Mn = 5000 g mol^−1^), FDA-approved and very common biopolymer in drug delivery [38] was used also for DAS encapsulation. The average size, size distribution (PdI) and Z-potential of DAS-loaded CHO/PA NPs and DAS-loaded PLA NPs, loading efficiency (LE) and encapsulation efficiency (EE) are listed in Table 3. The loading of the NPs with DAS did not lead to an increase of the R_H_. NPs of 100 nm can be efficiently taken up into cells by endocytosis [39,40], and the size of DAS-loaded CHO/PA NPs fell within this range. The hydrophobic character of both polymers leads to an optimal LE, while the formulation procedure was better optimized for the CHO/PA copolymer (see EE in Table 2). 

**Figure 4:**
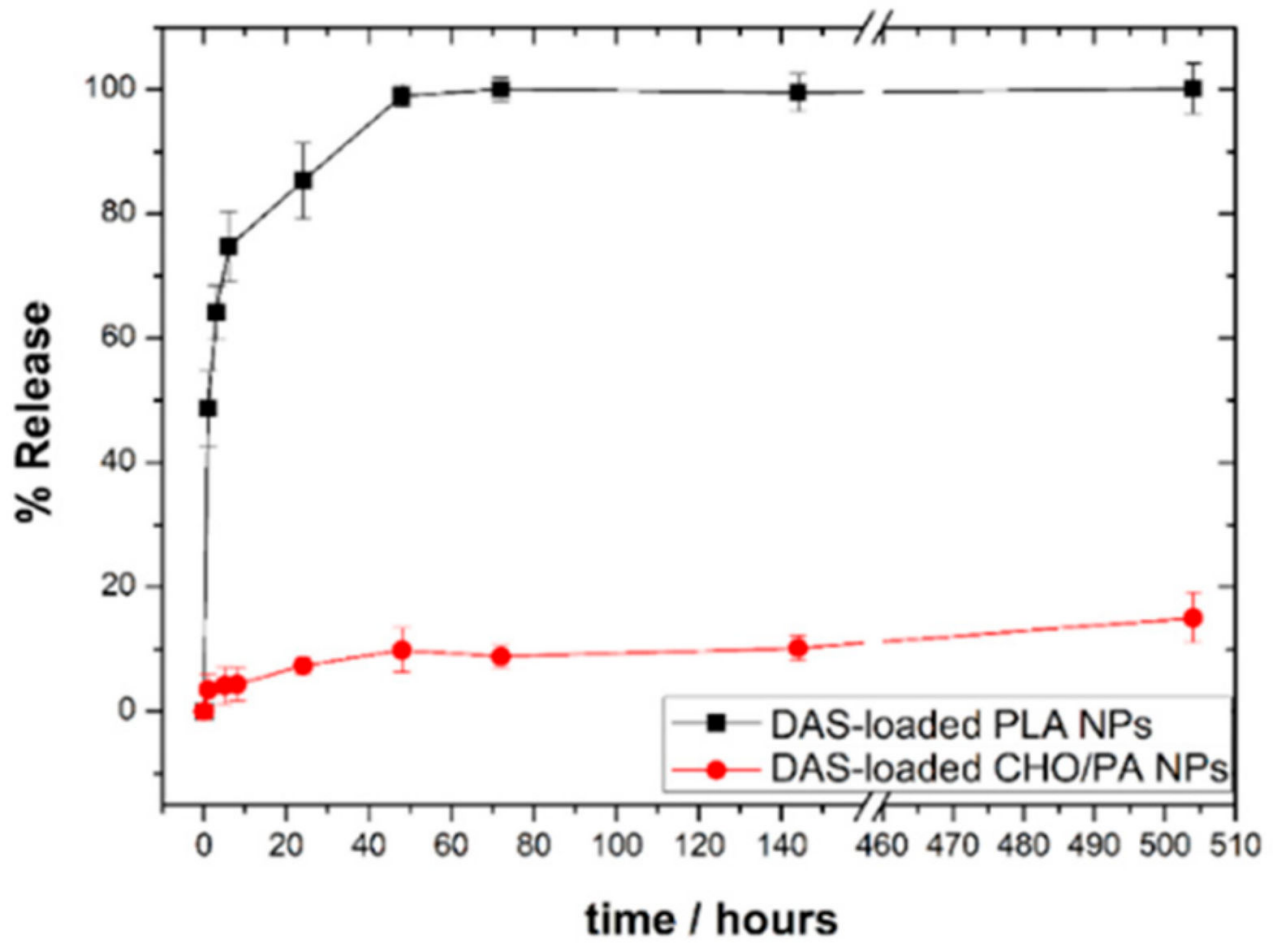
In vitro release profiles of DAS-loaded CHO/PA NPs and DAS-loaded PLA NPs at pH 7.4. Data are expressed as mean ± s.e.m. from at least three independent experiments.

The in vitro release of DAS-loaded NPs was studied in PBS (pH 7.4). As shown in Figure 4, DAS release kinetics showed an significant initial burst release and a sustained delivery over 60 h for DAS-loaded CHO/PA NPs. Unlike, a sustained deliver over weeks were observed for DAS-loaded PLA NPs. The release behavior of the different devices under investigation is related to the structural differences. The high crystallinity of the PLA NPs could hamper the diffusion phase release and affect the DAS delivery in a first step. The faster degradation of CHO/PA could explain the sustained release of DAS over hours. Complete target inhibition for a prolonged period increase efficacy of kinase inhibitors [41]. In this context, a significant initial burst release should be of crucial importance for kinase inhibition. The continuous and maintained delivery of DAS over hours can potentially augment the efficacy by increasing the exposure of the compound to the cancer cell maintaining the inhibition of the protein. Finally, release kinetics of DAS-loaded CHO/PA NPs at pH 5 is shown in Figure S5 in the Supplementary Materials. As it was expected, a very quick release of DAS was observed under acid environments [34].

Encapsulation of DAS on polymeric micelles offers a quicker sustained drug release (over 80 h) and smaller average size of the nanocarriers [26]. However, the low LE and poor stability limits their development for clinical purposes. PGA and PLA modification of carbon nanotubes were reported as a prerequisite for enhancement of the in vitro therapeutic efficacy of DAS against U-87 glioblastoma [30]. Albumin NPs were formulated as a DAS carrier to reduce DAS-induced endothelial hyperpermeability. The DAS-loaded NPs possessed equipotent anti-leukemia activity as DAS [29].

### 3.6. Stability and Compatibility in Blood of DAS-Loaded CHO/PA NPs

**Figure 5:**
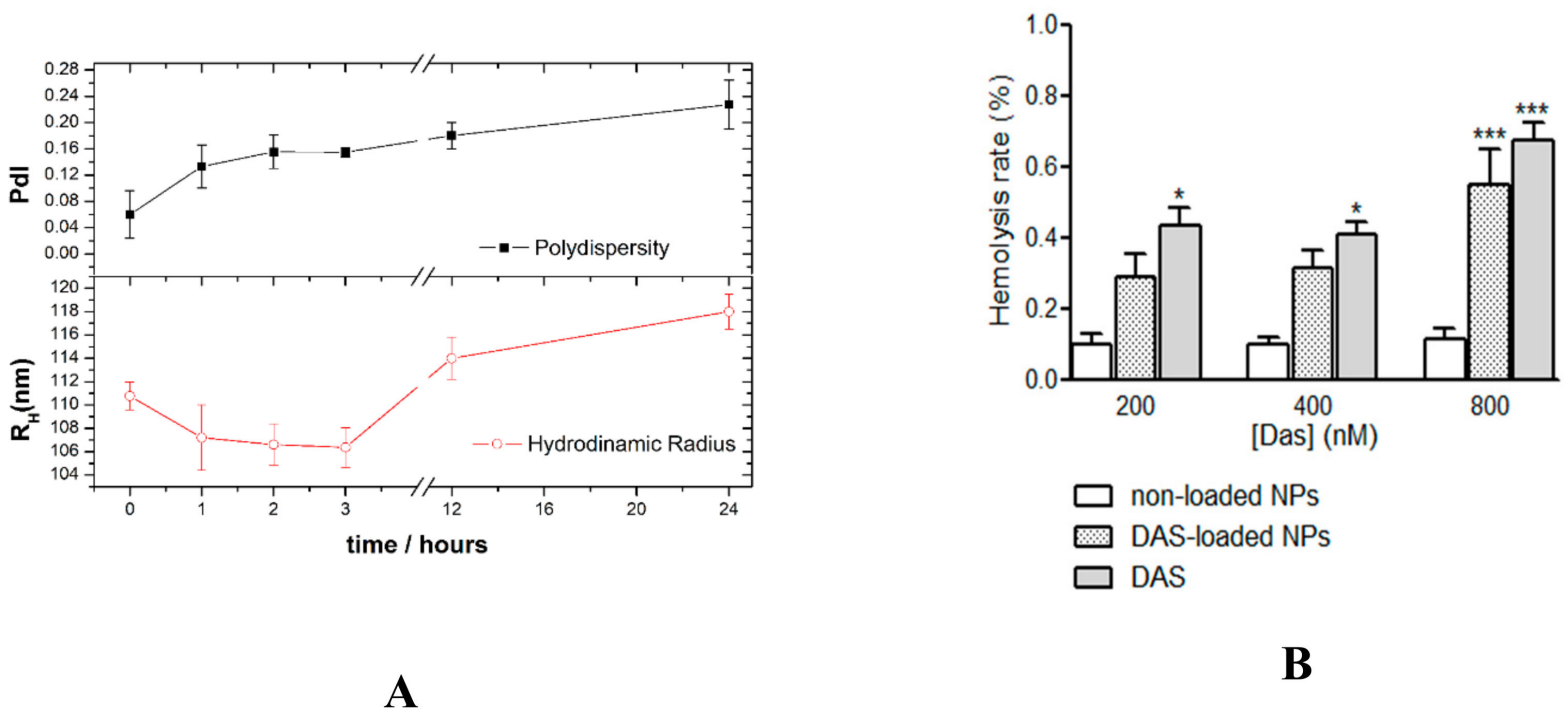
(**A**). Variation of hydrodynamic radio (R_H_) and polydispersity index (PdI) with the incubation time for DAS-loaded CHO/PA NPs. (**B**). Blood compatibility of CHO/PA NPs and DAS-loaded CHO/PA NPs. Data are expressed as mean ± s.e.m. from at least three independent experiments.

The parenteral administration of DAS-loaded NPs formulations could improve DAS bioavailability. The stability of DAS-loaded CHO/PA NPs was tested in vitro simulated biological media. The negligible changes on the R_H_ and PdI of the NPs suggested a high stability against aggregation in simulated biological media (Figure 5A). The slight increase in R_H_ and PdI should be related to the adsorption of a protein monolayer [42,43]. Steady levels of the drug compound in blood can increase the efficacy by exposing cancer cell to constant concentration of the compound with antiproliferative activity.

**Figure 6:**
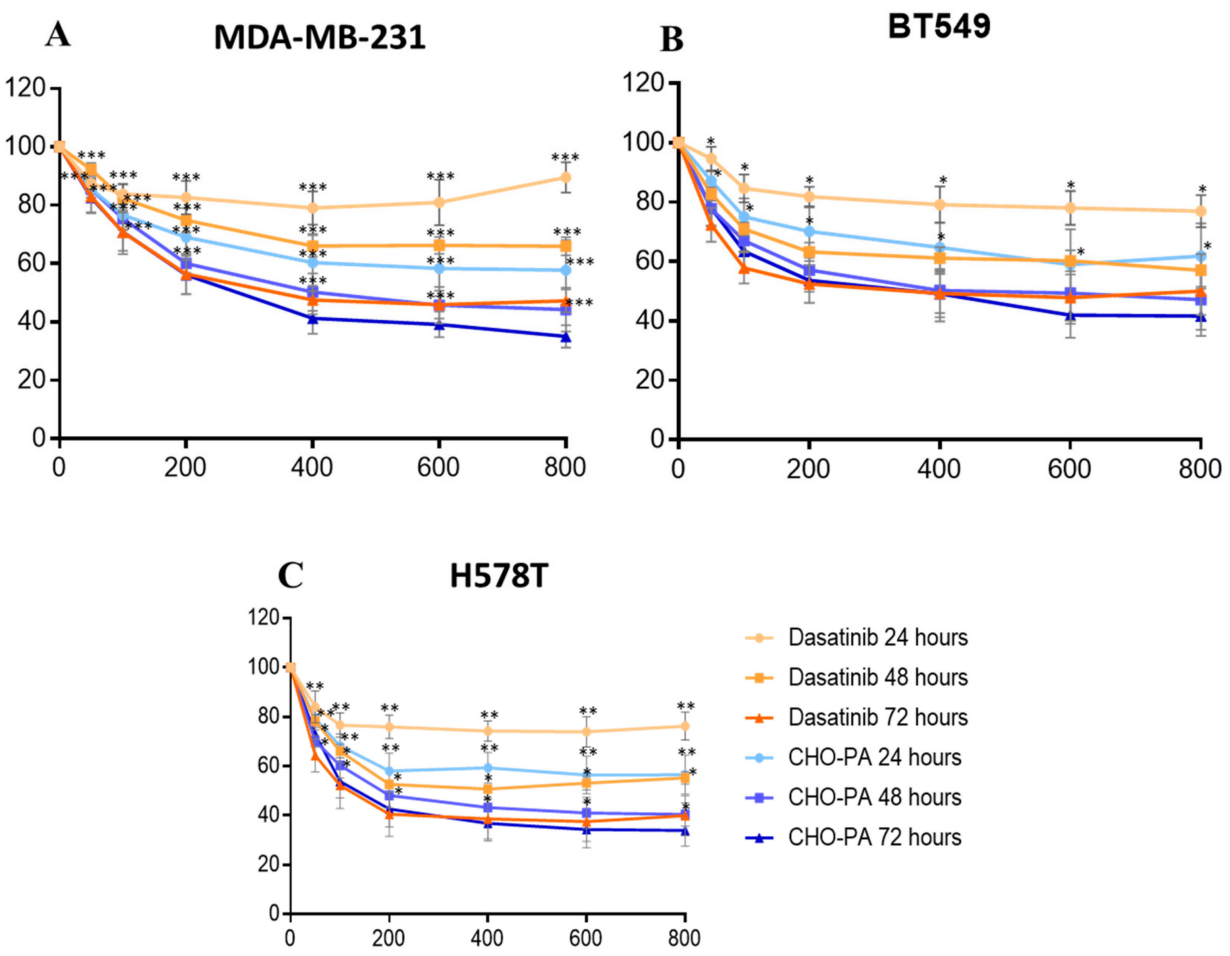
Time-course effect of DAS and DAS-loaded CHO/PA NPs (CHO/PA) on cell viability in (**A**). MDA-MB-231, (**B**). BT549 and (**C**). HS578T. Data are expressed as mean ± s.e.m. from at least three independent experiments. *p* < 0.05* *p* < 0.01** *p* < 0.001***.

The blood compatibility of CHO/PA NPs and DAS-loaded CHO/PA NPs were evaluated in terms of hemolysis (Figure 5B). A hemolysis rate lower than 5% is required for the materials having potential in biomedical applications. Human red blood cells were incubated in the presence of 200, 400, 800 nM DAS, 200, 400, 800 nM DAS-loaded CHO/PA NPs or the equivalent concentrations of non-loaded CHO/PA NPs (20, 40, 80 µg·mL^−1^, respectively) for 3 h. Saline solution and Triton 1% solution were used as a positive and negative control, respectively. Non-loaded CHO/PA NPs did not show a significant increase in the hemolysis degree at any of the concentration tested (lower than 0.1%). Compared with non-loaded NPs DAS slightly but significantly increase the hemolysis rate at the three concentrations studied, while DAS-loaded NPs only display a significant increase in the hemolytic rate at the highest concentration assayed (Figure 6B). It is noteworthy that in all cases the percentage of hemolysis rate was much lower than the 5% allowed according to ISO/TR 7406 (the critical safe biomaterials hemolytic ratio (5%)). Moreover, the increase in hemolysis rate seems to be more related to DAS presence rather than to CHO/PA NPs.

### 3.7. Antiproliferative Studies in Vitro

The cytotoxic effect of DAS-loaded CHO/PA NPs on tumoral cells were assayed by monitoring their ability to inhibit cell growth using MTT assays in several cell lines representative of triple negative breast cancer (TNBC), including MDA-MB-231, HS578T and BT549. Cells were treated with vehicle, DAS, or DAS-loaded CHO/PA NPs (0.2–0.8 µM) for 24, 48 and 72 h (Figure 6). Interestingly, the DAS-loaded CHO/PA NPs had a significant improved cytotoxic effect (IC_50_ = 0.4 μM at 48 h and 0.2 μM at 72 h) in comparison with free DAS with an IC_50_ > 0.8 µM at 48 h and 0.4 μM at 72 h. DAS-loaded PLA NPs were assessed on MDA-MB-231 and HS578T cells and no cytotoxic activity were observed (see Figure S6 in Supplementary Materials). These findings might indicate that the initial burst release is needed to observe an efficacy at early time points (24 h). The more efficient inhibition of the target might be achieved with the help of a sustained release over time. 

### 3.8. Cell Cycle Arrest and Apoptosis

**Figure 7:**
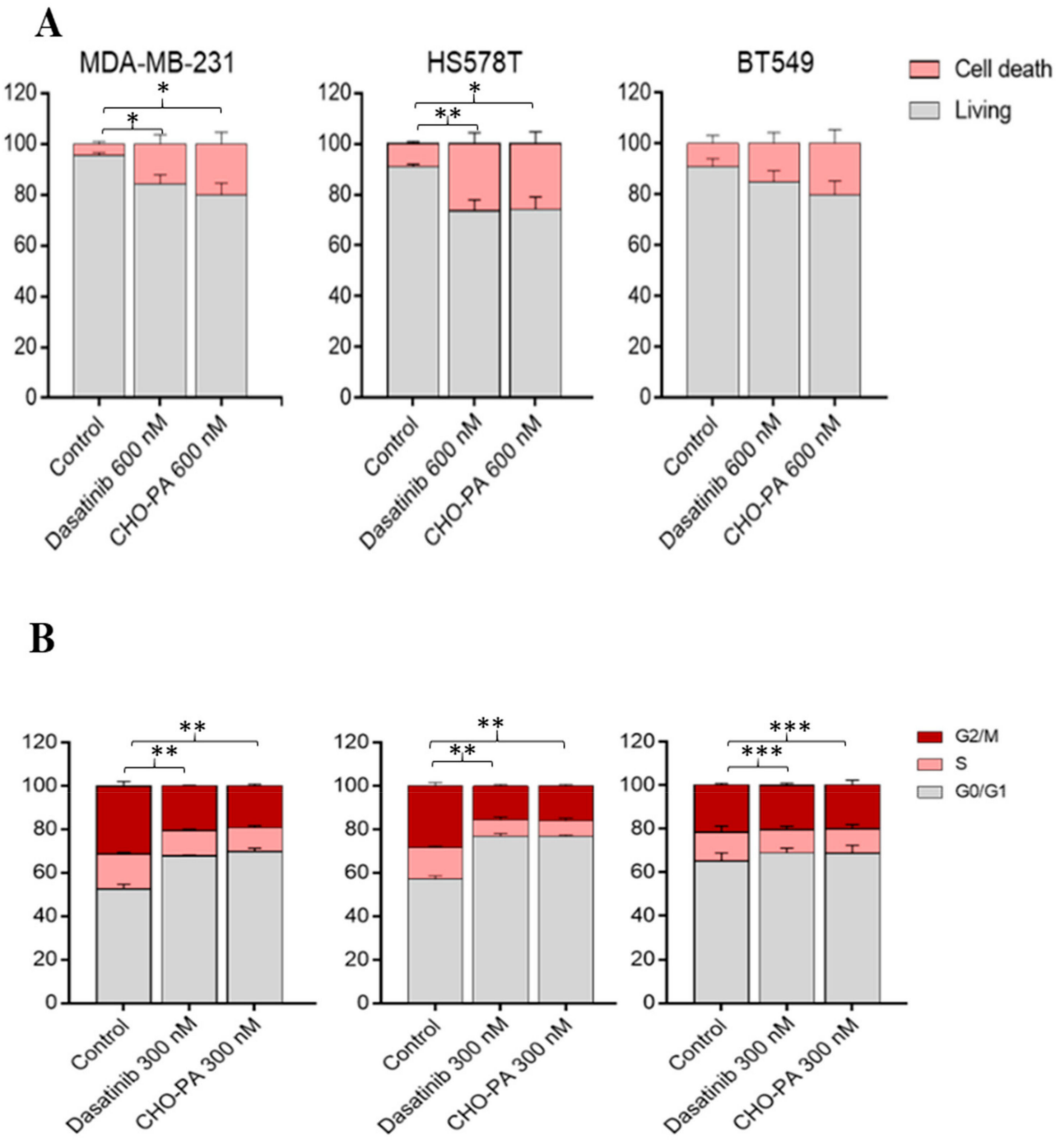
Effect of DAS and CHO/PA in TNBC cell lines. (**A**). Apoptotic effect of dasatinib and CHO/PA in MDA-MB-231, HS578T and BT549. Cells were stained with Annexin V after 72 h of treatment. (**B**). Effect in cell cycle. Graphical representation. Cells were treated with 600 nM of DAS and CHO/PA. Cell cycle progression was analyzed after 24 h of treatment by flow cytometry using propidium iodide DNA staining. Data are expressed as mean ± s.e.m. from at least three independent experiments. *p* < 0.05* *p* < 0.01** *p* < 0.001***.

Given the fact that DAS-loaded CHO/PA NPs inhibited cell proliferation in several breast cancer cell lines, we aimed to explore their mechanism of action. Thus, we treated the three TNBC cell lines, MDA-MB-231, HS578T and BT549 with vehicle, DAS, or DAS-loaded CHO/PA NPs for 24 h and, afterwards, we stained them with propidium iodide/RNase solution. Treatment with DAS-loaded CHO/PA NPs was able to slightly, but not reaching a statistically significant level, increase apoptosis in MDA-MB-231 and BT549, suggesting an increase in cell death (Figure 7A). On the other hand, administration of DAS-loaded CHO/PA NPs did not show any difference on the cell cycle phases, compared in free DAS. Globally, this data confirms that the NPs mediates their effect in the same manner as the compound in its free formulation (Figure 7B).

## 4. Conclusions

The biopolymer poly(cyclohexene phthalate) was successfully prepared from ROCOP and used, for the first time, to generate nanoparticles. The biodegradable copolymer is nontoxic, blood compatible and easily degradable in simulated biological media. The nanoparticles generated from this copolymer lead to encapsulate DAS in an efficient manner. A significant initial burst release but a sustained delivery over 60 h of the encapsulated DAS seems to be needed for a potential use in breast cancer therapy. In vitro cytotoxicity in different breast tumor cell models demonstrated the superior efficacy of the DAS-loaded CHO/PA NPs when compared to free DAS and DAS-loaded PLA NPs. In addition, no differences in the mechanism of action of the NPs were observed. The results indicate that DAS-loaded CHO/PA NPs might have potential in the development of DAS delivery systems against breast cancer. Further work is required to investigate their efficacy using in vivo models.

## Figures and Tables

**Table 1:** ROCOP of CHO and PA.

Entry	Conv. (%) ^2^	Polyester (%) ^2^	Mnexp ^3^	PdI ^3^
1 ^1^	100	95	3572	1.11
2 ^4^	100	94	3114	1.16
3 ^5^	100	95	2533	1.21
4 ^6^	100	98	2088	1.09

^1^ Reactions were carried out using [CHO]:[PA]:[Al]:[TBAB] = 200:200:1:1. ^2^ Determined by NMR. ^3^ Determined by GPC. ^4^ [CHO]:[PA]:[Al]:[TBAB] = 150:150:1:1. ^5^ [CHO]:[PA]:[Al]:[TBAB] = 100:100:1:1. ^6^ [CHO]:[PA]:[Al]:[TBAB] = 50:50:1:1.

**Table 2:** Hydrodynamic diameter (nm), polydispersity index (PdI) and Z-potential of CHO/PA NPs.

Entry	Mn_exp_	Average Size (nm)	PdI	Z-Potential (mV)
1	2088	100.0 ± 1.0	0.125 ± 0.02	−23.6 ± 1.74
2	2533	100.1 ± 0.7	0.123 ± 0.01	−21.9 ± 1.0
3	3114	113.7 ± 1.5	0.120 ± 0.03	−26.9 + 0.54
4	3572	107.3 ± 1.5	0.142 ± 0.04	−26.3 ± 0.8

**Table 3:** Full characterization of DAS-loaded CHO/PA NPs and DAS-loaded Polylactide NPs (PLA NPs): Average size, Polydispersity (PdI), Encapsulation efficiency (EE), loading efficiency (LE) and release percentage (RE) of DAS. Data are expressed as mean ± s.e.m. from at least three independent experiments.

Formulation	Average Size (nm)	PdI	Z-value (mV)	RE (%) (72 h)	EE (%)	LE (%)
CHO/PA NPs	104.5 ± 0.8	0.20 ± 0.01	32.1 ± 1.8	99.9 ± 2.0	96.7 ± 0.8	10.1 ± 0.4
PLA NPs	179.3 ± 3.8	0.20 ± 0.03	26.9 ± 1.74	8.8 ± 1.9	83.3 ± 2.5	11.7 ± 3.6

## Supplementary Materials

The following are available online at https://www.mdpi.com/2079-4991/9/9/1208/s1. General procedure and equipment, ^1^H-NMR spectrum of CHO/PA, TGA, DSC and SAXS/WAXS analysis of CHO/PA NPs are included. Figure S1 NMR of CHO/PA (Entry 4, Table 1); Figure S2. TGA analysis of raw material and NPs; Figure S3 DSC traces of NPs and in comparison with raw materials; Figure S4. SAXS and WAXS profiles of CHO/PA raw material and CHO/PA Nps; Figure S5. DAS-release profiles of DAS-loaded CHO/PA NPs at pH = 5; Figure S6. Effect of DAS and DAS-loaded PLA NPs on cell viability in MDA-MB-231 and HS578T.
