# Supplementary material for: Poly(Cyclohexene Phthalate) Nanoparticles for Controlled Dasatinib Delivery in Breast Cancer Therapy
Source: Nanomaterials (Basel). 2019 Aug 27;9(9):1208. doi: 10.3390/nano9091208 (PMC6780527; doi:10.3390/nano9091208)
Supplement: Supplementary file 1 [file nanomaterials-09-01208-s001.pdf]

ELECTRONIC SUPPORTING INFORMATION FOR

Poly(cyclohexene phthalate) nanoparticles for controlled Dasatinib delivery in breast cancer therapy

Enrique Niza,[a] Cristina Nieto-Jiménez,[b] María del Mar Noblejas-López,[b] Iván Bravo,[c] José Antonio Castro-Osma,[a] Felipe de la Cruz,[d] Marc Martínez de Sarasa Buchaca,[d] Inmaculada Posadas,[e] Jesús Canales-Vázquez,[f] Agustín Lara-Sanchez,[d] Daniel Hermida-Merino,[g] Eduardo Solano,[h] Alberto Ocaña[b]* and Carlos Alonso-Moreno[a]*

[a] Dpto. Inorgánica, Orgánica y Bioquímica. Facultad de Farmacia de Albacete, UCLM, 02071-Albacete,Spain.

[b] Oncología traslacional and CIBERONC, Unidad de Investigación del Complejo Hospitalario Universitario de Albacete. 02071-Albacete, Spain

[c] Dpto. Química Física. Facultad de Farmacia de Albacete, UCLM, 02071-Albacete, Spain.

[d] Dpto. Inorgánica, Orgánica y Bioquímica. Facultad de Ciencias y Tecnologías Químicas de Ciudad Real, UCLM, 13075-Ciudad Real, Spain

[e] Unidad Asociada Neurodeath CSIC-UCLM, Dpto. de Ciencias Médicas, Facultad de Farmacia, Universidad de Castilla-La Mancha, Campus Universitario de Albacete, 02071-Albacete, Spain.

[f] Instituto de Energías Renovables, Campus Universitario, Albacete-02071, Spain.

[g] Netherlands Organisation for Scientific Research (NWO), DUBBLE@ESRF, Grenoble, 38000, France.

[h] NCD beamline, ALBA Synchrotron Light Source, Carrer de la Llum 2-26, 08290 Cerdanyola del Vallès, Spain.

General procedures an equipment

All manipulations for the synthesis of CHO/PA were performed under nitrogen, using standard Schlenk techniques. Toluene was pre-dried over sodium wire and distilled under nitrogen from sodium. Deuterated solvents were stored over activated 4 Å molecular sieves and degassed by several freeze-thaw cycles. ^{1}H NMR spectra were recorded on a Varian Inova FT-500 spectrometer and referenced to the residual deuterated solvent. $AlEt_3$ 1M solution (Sigma-Aldrich) was used as received. Cyclohexene oxide (Sigma-Aldrich) was dried over CaH_2, distilled under *vacuo* and stored in the glovebox. Phthalic anhydride (Sigma-Aldrich) and Poly(L-lactide) (Sigma-Aldrich) were sublimed three times and stored in the glovebox. Aluminum compounds were prepared according to literature procedures.[1]

Solution polymerisation. In the glovebox, aluminum complex (20 μmol), Bu4NBr (20 μmol) and phthalic anhydride (4 mmol) were placed into a 10 mL Schlenk equipped with

[1] A. Otero, A. Lara-Sánchez, J. Fernández-Baeza, C. Alonso-Moreno, J. A. Castro-Osma, I. Márquez-Segovia, L. F. Sánchez-Barba, A. M. Rodríguez and J. C. García-Martínez, Organometallics, 2011, 30, 1507; J. A. Castro-Osma, C. Alonso-Moreno, M. V. Gómez, I. Márquez-Segovia, A. Otero, A. Lara-Sánchez, J. Fernández-Baeza, L. F. Sánchez-Barba and A. M. Rodríguez, Dalton Trans. 2013, 42, 14240

a small stir bar. Toluene (2 mL) was added and the reaction mixture was stirred. Then, cyclohexene oxide (4 mmol) was added and the reaction mixture was placed in a pre-heated oil bath at the desired temperature. After the appropriate time, a small aliquot was taken from the reaction mixture for NMR analysis to determine the monomer conversion and process selectivity. The viscous mixture was then dissolved in a minimum amount of dichloromethane or toluene and precipitated into an excess of MeOH or pentane. The polymer was then dried and collected as a white or light-yellow solid typically in 80-85% yield.

Size exclusion chromatography. Gel permeation chromatography (GPC) measurements were performed on a Polymer Laboratories PL-GPC-220 instrument equipped with a TSK-GEL G3000H column and an ELSD-LTII light-scattering detector. The GPC column was eluted with THF at 50 °C at a flow rate of 1 mL min^{-1} and was calibrated using eight monodisperse polystyrene standards in the range 580–483000 Da.

Thermal analysis. Thermogravimetric analysis for the devices was performed on a TGA instrument (model TGA-Q50). The heating rate for the sample was 10°C/min and the nitrogen flow rate was 60 mL/min.

Differential scanning calorimetry (DSC). DSC curves were obtained under N_2 atmosphere in a TA Instrument (model DSC-Q20). Samples were weighed into aluminum crucibles with 5 mg of sample and subjected to two heating cycles at a heating rate of 10°C/min.

Electron microscopy. The morphology of the nanoparticles was characterized by scanning electron microscopy (SEM) on a Jeol 6490 LV electron microscope equipped with an EDS detector (Oxford INCA Energy). Specimens were coated with Au-Pt using a SC7620-Quorum Technologies sputter coater to avoid charging-up problems on the specimen surface and to achieve better image resolution.
Higher resolution images were obtained by transmission electron microscopy (TEM) on a Jeol JEM 2100 electron microscope operating at 200 kV and equipped with an EDS detector (Oxford INCA Energy). TEM specimens were prepared by dispersing the polymers in distilled water and depositing few drops of the suspension on to lacey carbon-coated copper grids (EMS). The images were recorded under low-dose conditions to minimize beam damage and analyzed using Digital Micrograph™ software from Gatan.

Hydrodynamic diameter and Z-potential measurements. The average sizes, polydispersities and Z-potentials of the formulations were measured using a Zetasizer Nano ZS (Malvern Instruments). Specifications: number of measurements, 5; medium viscosity, 1.054 cP; refractive index, 1.33; scattering angle, 173°; α = 633 nm; temperature, 25°C. Data were analyzed using the multimodal number distribution software included in the instrument.

SAXS/WAXS experiments. Simultaneous SAXS/WAXS experiments were performed at NCD station of Alba synchrotron in Cerdanyola del Valles. The sample to SAXS

detector distance was ca. 2605 mm using a wavelength of 0.99 Å. A Dectris-Pilatus 1M detector with a resolution of 981×1043 pixels and a pixel size of 172 × 172 μm was employed to record the 2D SAXS scattering patterns. Standard corrections for sample absorption and background subtraction were performed. The data were normalized to the intensity of the incident beam (in order to correct for primary beam intensity fluctuations) and were corrected for absorption, background scattering. The scattering patterns from AgBe were used for the calibration of the wavevector scale of the scattering curve.
WAXS patterns were collected using a Rayonix Lx 255-HS with a pixel size of 44×44 μm and active area of 85 × 255 mm^2. The wavenumber $q = 4\pi/\lambda \sin\theta$ scale for WAXS experiments scale calibration has been achieved by Cr_2O_3.

Encapsulation parameters. Loading efficiency (LE) and encapsulation efficiency (EE) of DOX were calculated according to the following equations:

$$LE\ \% = (\text{weight of encapsulated DOX (mg)})/(\text{weight of total (DOX encapsulated+scaffold weight)(mg)}) \times 100\%$$

$$EE\ \% = (\text{weight of encapsulated DOX (mg)})/(\text{weight of DOX feeding (mg)}) \times 100\%$$

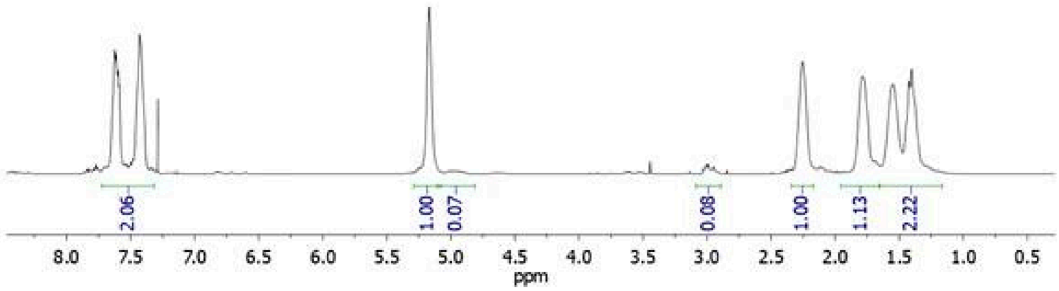

Figure S1. NMR of CHO/PA (Entry 4, Table 1)

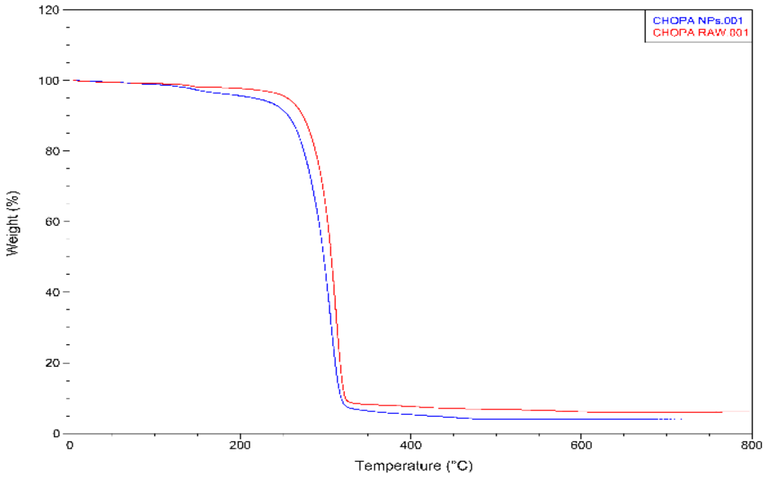

Figure S2. TGA analysis of raw material and NPs.

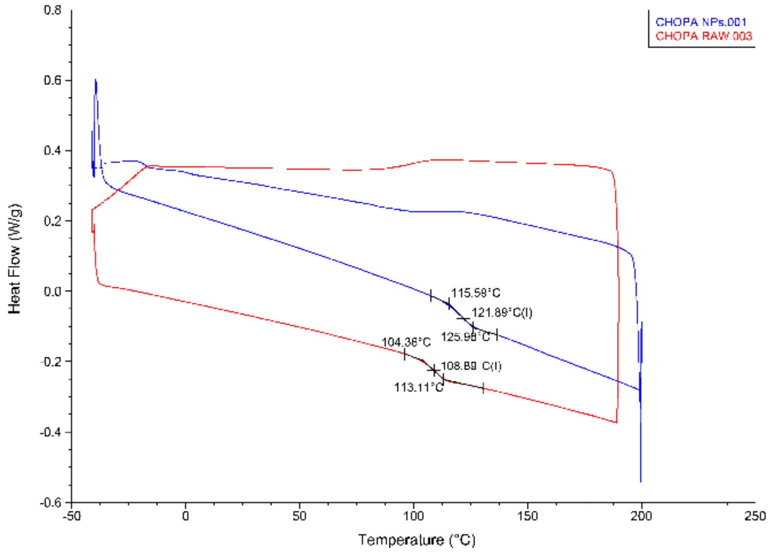

Figure S3 DSC traces of NPs and in comparison with raw materials.

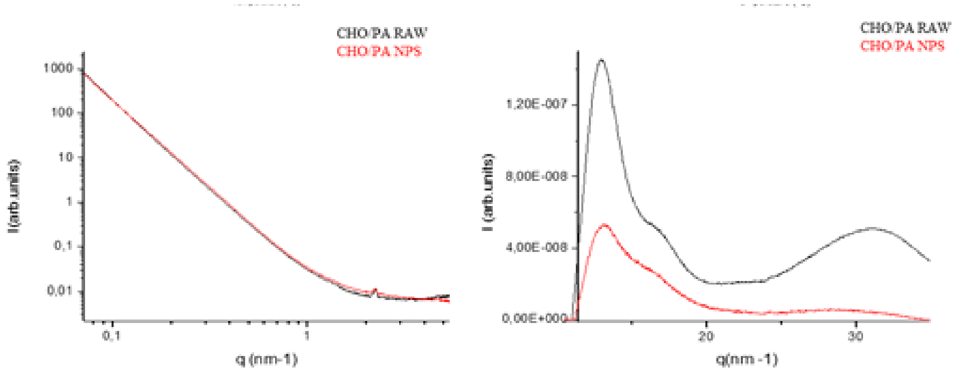

Figure S4. SAXS and WAXS profiles of CHO/PA raw material and CHO/PA Nps.

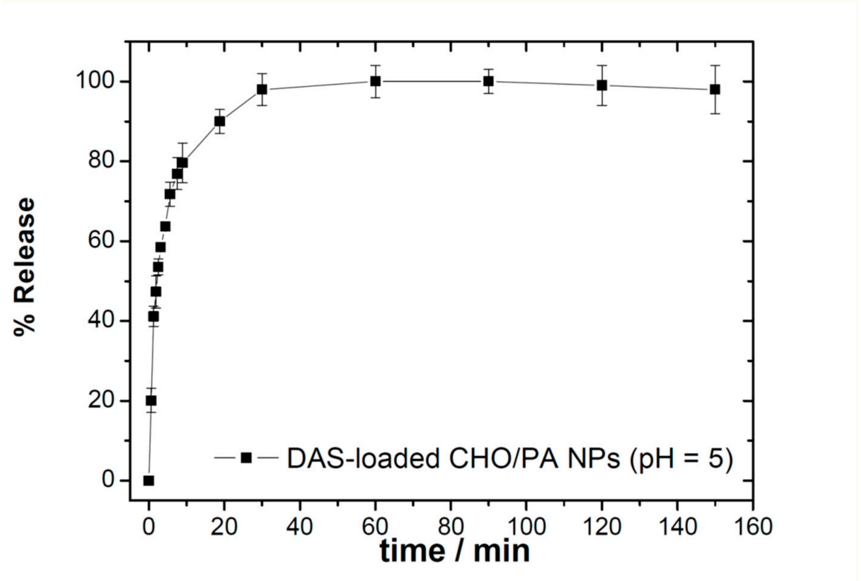

Figure S5. DAS-release profiles of DAS-loaded CHO/PA NPs at pH = 5.

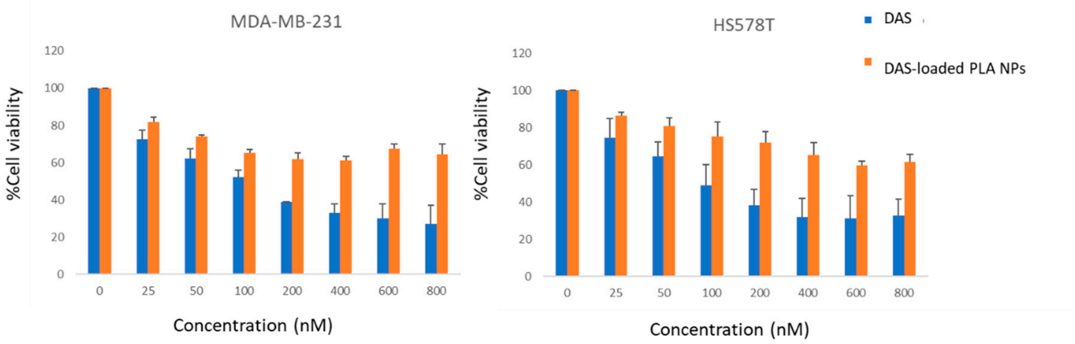

Figure S6. Effect of DAS and DAS-loaded PLA NPs on cell viability in MDA-MB-231 and HS578T. Data are expressed as mean ± s.e.m. from at least three independent experiments.
